# A case report of bilateral lateral ventricle calcified pseudoneoplasm of the neuraxins

**DOI:** 10.1186/s41016-023-00344-1

**Published:** 2023-10-18

**Authors:** Xiaolong Qiao, Yinan Chen, Ying Ji, Chaoshi Niu, Chuandong Cheng

**Affiliations:** 1 Anhui University of Science and Technology, Huainan, Anhui 232001 People’s Republic of China; 2 Department of Neurosurgery, The First Affiliated Hospital of USTC, Division of Life Sciences and Medicine, University of Science and Technology of China, 1st Tianehu Road, Hefei, Anhui 230031 People’s Republic of China

**Keywords:** CAPNON, Imaging, Pathology, Operation

## Abstract

**Background:**

Calcifying pseudoneoplasm of the neuraxis (CAPNON) is indeed a rare central nervous system lesion that can occur in central nervous system (CNS). Due to its infrequency and limited literature reports, it is challenging to diagnose and manage CAPNON.

**Case presentation:**

In this intriguing study, we embarked on a quest to uncover the story of a 16-year-old girl who experienced bothersome headaches. Through advanced imaging techniques like computed tomography (CT) and magnetic resonance imaging (MRI), we glimpsed a delicate calcified growth within the lateral ventricles’ posterior horn. Motivated by our unwavering commitment to solving mysteries, we embarked on a surgical journey that not only freed the young patient from her ailment but also shed light on the true nature of her puzzling adversary—a remarkable CAPNON.

**Conclusions:**

For patients with CAPNON who have multiple or non-respectable lesions, the primary goal is to alleviate symptoms. After alleviating the symptoms with partial resection, close monitoring of any residual lesions is essential. If there is no evidence for disease progression, a strategy of continued close observation is appropriate.

## Background

In 1978, researchers Rhodes and Davis made a significant discovery about a condition called CAPNON [1]. CAPNON involves complex fibrous structures within the brain, which have intrigued experts and earned the name “brain stones.” The causes of this condition are still not fully understood, with different theories proposing reactive cell growth [2], transformative changes [3], or even the development of a unique type of tumor [4]. In medical literature, CAPNON is generally considered to be a non-cancerous condition, but it is accompanied by inflammation resembling delicate granulomas that surround the intricate calcified patterns. Unfortunately, the symptoms experienced by those affected stem from compression and the resulting inflammatory responses in the surrounding tissue [5].

The interaction between CAPNON and complex partial seizures is a complex dance, where the precise placement of these calcified formations in the temporal lobe dictates the unfolding of events [5–7]. Their influence on the spinal cord leads to symptoms of compression, serving as a vivid reminder of their impact on our physical well-being [8]. However, a truly remarkable occurrence awaits those fortunate enough to witness CAPNON gently impinging on the revered oculomotor nerve, restricting the eye’s freedom of movement [9]. This celestial spectacle represents the groundbreaking observation of CAPNON within the bilateral lateral ventricles, a revelation that expands the horizons of our understanding.

In this captivating narrative, we unveiled hidden truths, igniting curiosity about CAPNON as a potential explanation for enigmatic calcifying CNS lesions. United by a thirst for knowledge, we sought to illuminate effective approaches, unlocking CAPNON’s remarkable potential.

## Case presentation

A 16-year-old girl was hospitalized due to persistent headaches that had been occurring for more than a month. Alongside the headaches, she experienced nausea and vomiting. Importantly, she had no prior history of such symptoms, and there were no limb convulsions present. Following a thorough consultation and neurological examination, the doctors ruled out epilepsy and intracranial hypertension as potential explanations for the symptoms. To further investigate the condition, imaging studies were conducted.

**Fig. 1:**
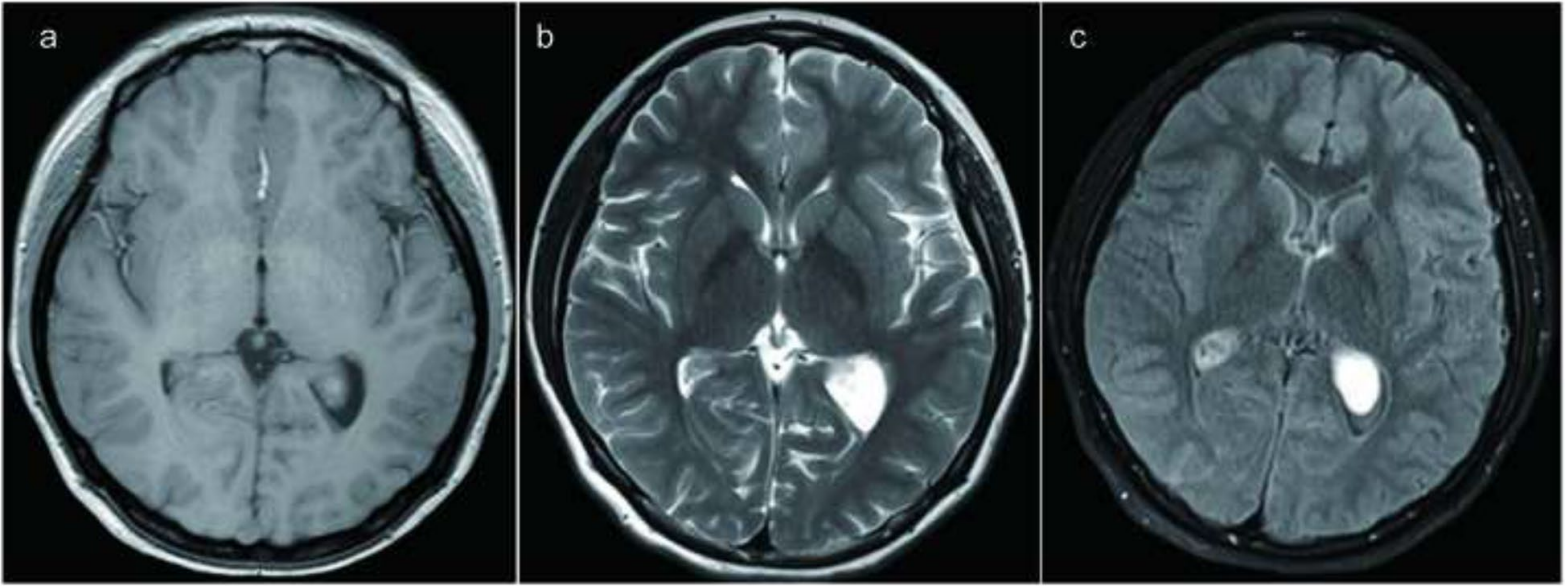
Initial magnetic resonance imaging. **a** Low-intensity area was evident in the bilateral lateral ventricles on T1-weighted images. **b** Low intensity area was seen on T2 weighted images. **c** Low intensity area was seen on fluid attenuated inversion recovery images

**Fig. 2:**
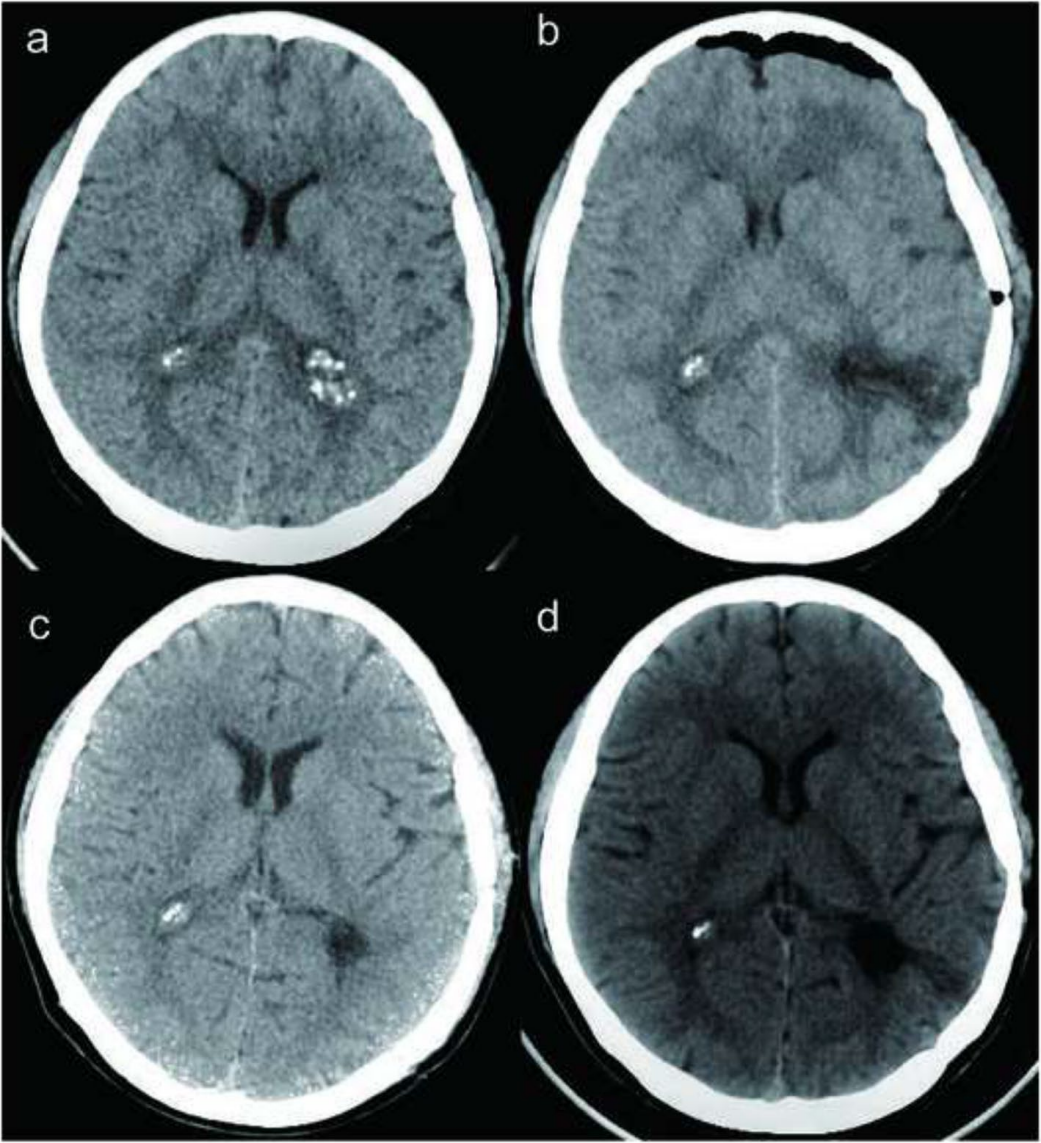
Presents CT scans of the head before and after surgery. The high-density lesion on the left side was successfully removed without any residual calcification. **a** The preoperative head CT scan. **b**–**d** Head CT scans taken at 3 days, 1 month, and 27 months after surgery, respectively. It is evident that the left mass was completely eradicated with no signs of recurrence, while the right mass remained unchanged in size

**Fig. 3:**
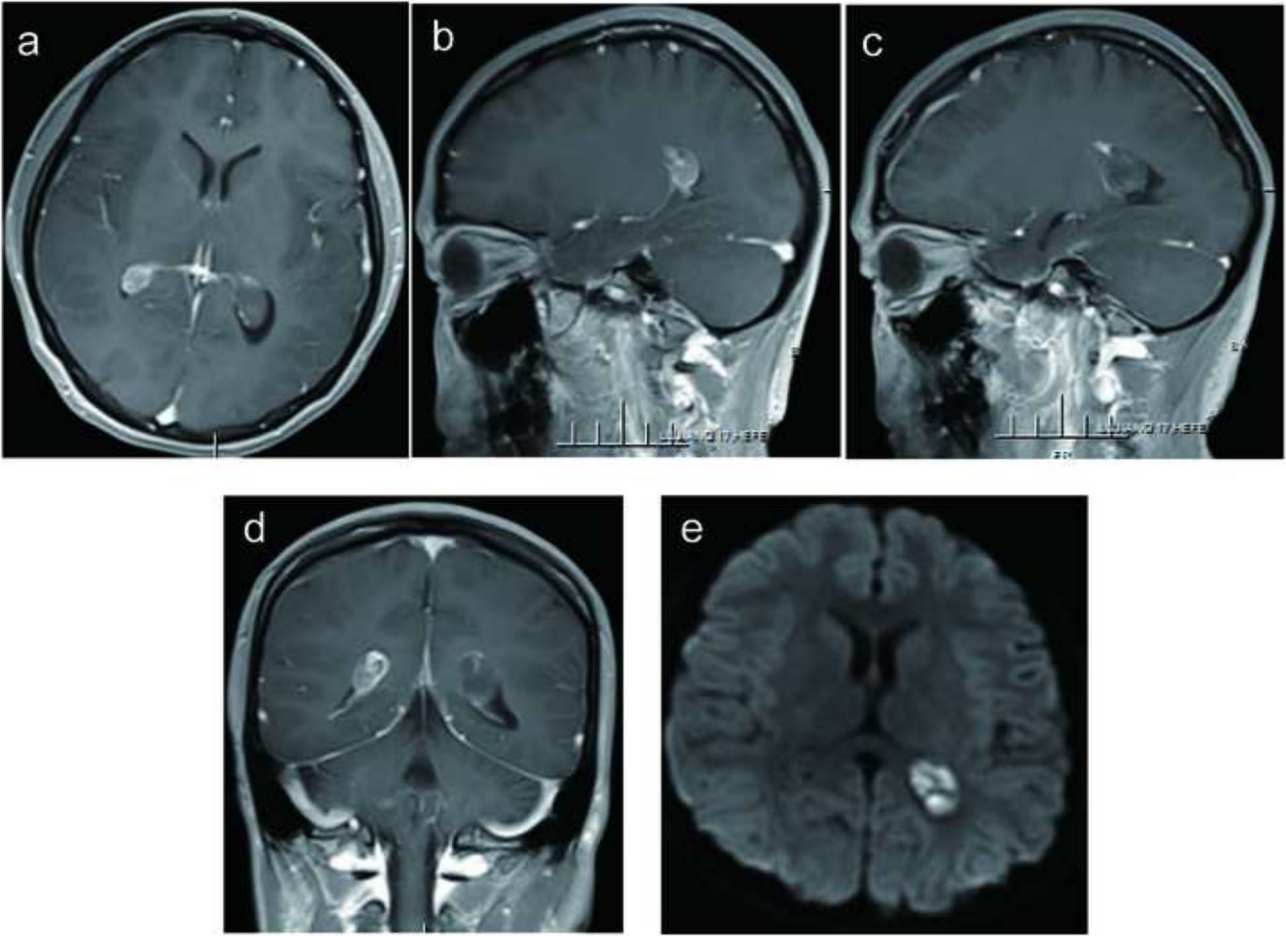
MRI demonstrating multiple CAPNONs. In T1-weighted post-contrast MRI images, there is an absence of contrast enhancement observed in the bilateral lateral ventricles. Please refer to the following horizontal (**a**), anomalous (**b**, **c**), and coronal (**d**) images; diffusion-weighted imaging (DWI) sequence showed low signal shadow in bilateral lateral ventricle (**e**)

A magnetic resonance imaging (MRI) scan was performed, revealing a dark hypointense lesion on both T1- and T2-weighted images. Notably, there was no swelling caused by leaking fluid from blood vessels (vasogenic edema). The MRI images displayed the presence of a choroid plexus yellow granuloma, as illustrated in Fig. 1a–c. To confirm the diagnosis, a computed tomography (CT) scan of the head was carried out. This scan confirmed the presence of brain lesions located in the bilateral lateral ventricles, as shown in Fig. 2a. The T1-weighted images taken after administering a contrast agent did not reveal any contrast enhancement in the bilateral lateral ventricles, as depicted in Fig. 3a–e. Based on the imaging results and the symptoms observed in the patient, we concluded that the cause of her condition was a brain lesion known as a choroid plexus yellow granuloma, located in the bilateral lateral ventricles. The absence of contrast enhancement in the post-contrast images indicated that the lesions were not actively growing or receiving an increased blood supply.

After carefully assessing the patient’s symptoms and examining the imaging results, it was clear that medication alone was not effectively treating the condition. Therefore, a surgical procedure was chosen as the next course of action. The primary objective of the surgery was to alleviate the patient’s headaches while also aiming to obtain a definitive diagnosis. For the surgical approach, the decision was made to focus on the left side and remove the lesion in the left lateral ventricle with the help of MRI guidance. This choice was based on several considerations. Firstly, attempting to completely remove the lesion from both sides through a single approach would have been challenging. Therefore, a unilateral surgical approach was preferred. Secondly, since the diagnosis was still uncertain before the surgery, it was important to clarify it while prioritizing maximum resection on the left side to relieve the patient’s headache symptoms. Finally, the lesion on the right side was relatively minor, and addressing both sides simultaneously would have posed additional surgical risks.

After the surgery, the patient experienced a significant reduction in their headache symptoms, indicating a positive outcome. To assess the patient’s neurological condition, doctors performed preoperative and postoperative evaluations using the National Institutes of Health Neurological Impairment Score (NIHSS) scale. These assessments did not reveal any neurological deficits, indicating that the surgery successfully preserved neurological function. The patient’s diligent postoperative care played a vital role in ensuring a complication-free recovery.

**Fig. 4:**
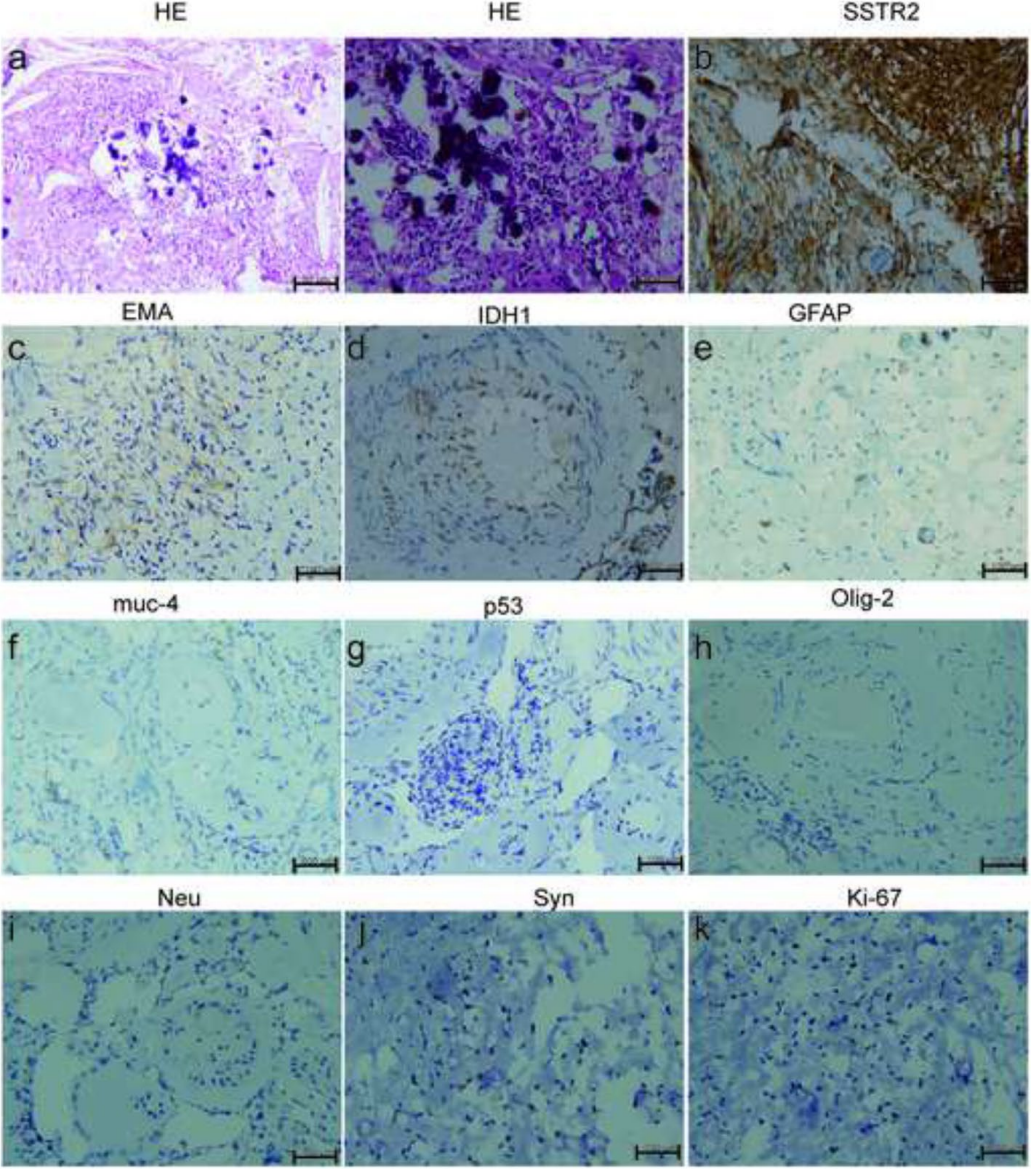
Photomicrographs of surgical specimens. The lesion consists of amorphous lamellar calcification, with eosinophilic matrix in the background on hematoxylin–eosin (HE) staining (**a**). **b**–**k** SSTR2 ( +), EMA (a few cells +), IDH1 ( −), GFAP ( −), muc-4 ( −), p53 ( −), 0lig-2 ( −), Neu ( −), Syn (partial +) and Ki-67 (+ 1%)

During the surgery, a tissue sample was obtained and subjected to histopathological examination. The hematoxylin–eosin (HE) staining examination revealed a hypo cellular nodule with calcium deposits. The margins of the lesion showed infiltration of lymphocytes or plasma cells (Fig. 4a). Immunohistochemical analysis further demonstrated the presence of somatostatin receptors 2 (SSTR2 +), with a few cells testing positive for epithelial membrane antigen (EMA). However, markers such as IDH1, GFAP, muc-4, p53, Olig-2, and Neu were negative. Synaptophysin (Syn) staining showed partial positivity. The Ki-67 proliferation index, which indicates the rate of cell division, was determined to be 1% (Fig. 4b–k). These findings provide important insights into the characteristics of the lesion.

After the surgical resection of the lesion in the left lateral ventricle, postoperative CT scans confirmed the complete removal of the calcified lesion on the left side. However, calcification was still present on the right side of the ventricle. Over the 27-month follow-up period, subsequent CT scans showed no recurrence of the lesion on the left side, indicating a successful resection. The calcification on the right side remained stable without significant enlargement (Fig. 2), suggesting it was not actively growing or causing immediate concern. However, regular monitoring will be necessary to track any changes in the calcification over time.

The surgery effectively alleviated the patient’s headaches and did not result in any neurological impairment. The analysis of the tissue sample provided important information about the nature of the lesion, and the subsequent imaging confirmed the successful removal of the lesion on the left side. It is crucial to maintain regular monitoring to ensure the condition remains stable and to detect any potential recurrence or advancement in the future.

## Discussion

CAPNONs are rare lesions in the central nervous system that can occur in both adults and children. While partial removal of CAPNONs in the spinal cord has been reported [8], there have been no documented cases of partially removing intracranial lesions. After considering the patient’s medical history and test results, we decided to perform surgery to relieve the patient’s headache symptoms. To minimize risks, we chose a less aggressive approach instead of removing tumors from both sides. Our main focus was on surgically removing the larger lesion on the left side.

Follow-up assessments conducted for 27 months after the surgery showed that the right lateral ventricle lesion did not grow, and there was no recurrence in the left-sided lesion. When dealing with bilateral CAPNON, our clinical strategy involves treating one side of the lesion while closely monitoring the other side to reduce surgical risks.

In this study, we were surprised to find that the swelling in CAPNON lesions was mild compared to previous reports [10]. We believe this could be due to the small size and location of the lesion within the brain’s ventricles. The lesion’s position did not block blood flow significantly, resulting in less swelling and suggesting that it did not deeply affect the surrounding brain tissue [11].

Additionally, we identified some distinctive characteristics of bilateral lateral ventricular CAPNON lesions compared to unilateral ones [12]. Lesions within the ventricles tend to have less swelling, and bilateral multiple lesions are less likely to have calcifications compared to unilateral lesions. Moreover, if a lesion does not cause any symptoms, it can be safely managed without immediate treatment.

## Conclusion

In summary, CAPNON, characterized by bone-like components in intracranial lesions, was typically benign but could cause symptoms due to compression and inflammation. This case stressed the need to consider CAPNON as a differential diagnosis for calcified CNS lesions, highlighting the importance of accurate diagnosis for proper management. In situations where complete removal of multiple lesions was unfeasible in a single surgery, a selective approach proved more effective. Prioritizing symptomatic or larger lesions acknowledged the complexities in managing multiple lesions and offered a practical strategy.

## Data Availability

Not applicable.

## Abbreviations

CAPNON: Calcifying pseudoneoplasm of the neuraxis
CNS: Central nervous system
CT: Computed tomography
MRI: Magnetic resonance imaging
NIHSS: National Institutes of Health Neurological Impairment Score
HE: Hematoxylin–eosin
SSTR2: Somatostatin receptors 2
EMA: Epithelial membrane antigen
Syn: Synaptophysin
DWI: Diffusion-weighted imaging
